# Evaporimeter and Bubble-Imaging Measures of Sweat Gland Secretion Rates

**DOI:** 10.1371/journal.pone.0165254

**Published:** 2016-10-21

**Authors:** Jeeyeon Kim, Miesha Farahmand, Colleen Dunn, Zoe Davies, Eric Frisbee, Carlos Milla, Jeffrey J. Wine

**Affiliations:** 1 Cystic Fibrosis Research Laboratory, Stanford University, Stanford, California, United States of America; 2 Department of Pediatrics, Stanford University School of Medicine, Stanford, California, United States of America; 3 Department of Psychology, Stanford University, Stanford, California, United States of America; University of Alabama at Birmingham, UNITED STATES

## Abstract

Beta-adrenergically-stimulated sweat rates determined by evaporimetry or by sweat bubble imaging are useful for measuring CFTR function because they provide a near-linear readout across almost the full range of CFTR function. They differentiate cystic fibrosis (CF) subjects from CF carriers and carriers from controls. However, evaporimetry, unlike bubble imaging, appears to be unable to detect improved levels of CFTR function in G551D subjects taking the CFTR modulator ivacaftor. Here, we quantify the sensitivity of evaporimetry and bubble imaging methods for assessing low levels of CFTR-dependent sweat rates. To establish sensitivity, we did dose-ranging studies using intradermally injected [cAMP]_i_–elevating cocktails. We reduced isoproterenol/aminophylline levels while maintaining a high level of atropine to block muscarinic elevation of [Ca^2+^]_i_. We stimulated the same sets of glands for both assays and recorded responses for 20 min. In response to a 3-log dilution of the stimulating cocktail (0.1%), bubble responses were detected in 12/12 tests (100%), with 49% ± 3% of glands secreting to produce an aggregate volume of 598 nl across the 12, 20-min tests. This was ~5% of the response to full cocktail. Evaporimetry detected responses in 3/12 (25%) tests with an aggregate secretion volume of 175 nl. After stimulation with a still more dilute cocktail (0.03%), bubble imaging detected 15 ± 13% of glands secreting at a rate ~0.9% of the response to full cocktail, while zero responding was seen with evaporimetry. The bubble imaging method detected secretion down to aggregate rates of <0.2 nl/(cm^2^·min), or ~1/30^th^ of the average basal transepithelial water loss (TEWL) in the test subject of 4 g/m^2^·hr or 6.7 nl/(cm^2^·min). The increased sensitivity of bubble imaging may be required to detect small but physiologically important increases in secretion rates produced by CFTR modulators.

## Introduction

The eccrine sweat gland is a near-ideal organ for assessing the function of CFTR—the anion channel that causes cystic fibrosis (CF) when absent or defective [1]. Sweat is secreted by several million simple, tubular glands that express CFTR in both the absorptive duct and secretory coil. The glands are readily accessible and do not suffer from the secondary changes observed in many CF organs [2]. Elevated NaCl concentration in sweat was the earliest consistent method for diagnosing CF [3] and is now buttressed by decades of experience in virtually all CF centers worldwide. The sweat chloride level is also a sensitive indicator for detecting improved CFTR function in response to CFTR modulators [4], and recent exhaustive work to track sources of variation in the sweat test determined that most variation is due to variation of CFTR [5]. Sweat chloride values change as a log function of CFTR activity, providing a highly sensitive indicator of very low levels of CFTR function, but becoming increasingly less sensitive at higher values [6]. Hence, sweat chloride values for CF carriers are barely different than those of normal controls [7].

In 1984, Sato & Sato discovered that human eccrine sweat glands of people with CF failed to secrete to β-adrenergic agonists, but had apparently normal responses to cholinergic stimulation [8]. This complete absence of responding in the CF subjects suggested that the CF gene product (then unknown) was rate-limiting for β-sweat secretion, and this was confirmed by showing that CF carriers had half-normal β-sweat rates when those rates were expressed as ratios of their cholinergic sweat rates [9]. Thus, the response to a β-adrenergic cocktail that includes atropine to block cholinergic function is CFTR-dependent, and for simplicity has been termed ‘C-sweat’ [10]. Sweat secretion to muscarinic stimulation, which elevates [Ca^2+^]_i_, is termed M-sweat and is functional in CF subjects, and with appropriate controls the C/M sweat ratio provides a near-linear readout of CFTR function [6, 9–11]. The near-linearity of the C/M sweat secretion rate ratio over most of the range of CFTR function provides many advantages. Sweat rate measurement is also important because it is not yet certain if CFTR functions identically when involved in absorption (sweat duct) vs. secretion (sweat gland coil and most other organs affected in CF).

One limitation of measuring C-sweat rates is that they are much lower than M-sweat rates [8], in part because M-sweating is driven by basolateral, Ca^2+^-activated K^+^ channels, whereas the sweat coil appears to lack cAMP-activated K^+^ channels, so that C-sweat relies on the resting K^+^ conductance [12]. Furthermore, when CFTR function is very low, C-sweat rates can be equal or less than a set of subtractive factors, such as physical capacitance of the gland lumen and some fluid absorption by the duct, resulting in the absence of C-sweat at the duct orifice [6, 12]. This inherent limitation becomes critical when attempting to assess the effects of CFTR modifying compounds that are now being brought to the clinic [13–15], and emphasizes the need for determining the sensitivity and detection limits of assays that seek to measure partially compromised CFTR function. With this in mind we here compare the dynamic range of the two methods for measuring C-sweat rates with particular regard to their ability to detect low levels of CFTR function.

The evaporimeter is a probe with an open cylinder containing two stacked sensors that measure temperature and relative humidity at points ~4 mm apart vertical to the skin surface; the difference in water-vapor concentration is electronically converted and displayed as flux with units of g/(m^2^·h). Bubble imaging is achieved by placing an illuminated, oil-filled reservoir on the skin surface and digitally imaging the growth of spherical sweat bubbles as they form.

## Materials and Methods

### Subjects

This study was approved by the Institutional Review Board of Stanford University. All subjects were adults and written informed consent was obtained. Bubble imaging is well suited to n-of-one studies [10, 11]. We used a single male heterozygote subject (EB01) for dose-ranging experiments. This subject has tattooed sites (~1 mm dots) to facilitate gland identification and has been repeatedly tested in the past so that extensive reference data are available for the population of glands selected. To explore the basis of differences in transepidermal water loss (TEWL) measures across subjects, we also tested two female subjects, EB02 and EB03, who differed substantially in their baseline TEWL measurements.

### Measurement of sweat secretion rates from identified individual glands

**Fig 1:**
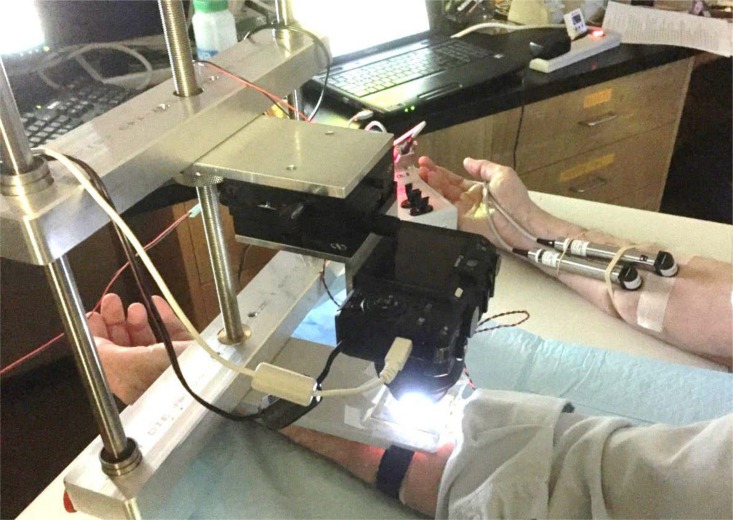
Sweat bubble imaging and evaporimetry. Here the left arm is being tested using sweat bubble imaging and the right with evaporimetry. The reservoir slides into the holder which is held in fixed position to the frame; the camera can be precisely positioned using micrometer drives. Two probes are used for evaporimetry: one stays on the same patch of skin throughout and serves as a control; the other is removed to allow for injections and then replaced. Two tattooed sites on each arm were used to ensure that the same populations of identified glands were sampled by each assay.

Previously described methods [10, 11] were modified to suit the present aim of comparing the sensitivities of evaporimetry and bubble imaging. Two tattooed sites on each volar forearm were selected for study. Left arm sites L2 and L3 were 4.6 cm apart with L2 12.5 cm from the wrist crease; R2 and R3 were 7.4 cm apart, with R2 13.7 cm from the wrist crease. All 4 sites were tested on a given day, with test days one week apart to allow for recovery from injection. One arm was used for evaporimetry and the other for bubble testing on each day, and each method was used on alternate arms at weekly intervals (**Fig 1**).

For the bubble imaging method, bubbles of sweat from identified single glands were captured in an oil layer, visualized by dye-partitioning, and digitally imaged at 30 sec intervals; sweat volumes and rates for identified glands were determined using ImageJ measurements of sweat bubbles as previously described [10]. Each site had 40–71 identified glands.

### Evaporimetry measurement of TEWL from areas with identified sweat glands

After completing the diagnostics procedure (cyberDERM, Inc. TEWL Technical Guide v. 1.03), two evaporimeter probes were placed on the volar surface of the forearm after lightly swabbing the sites with an alcohol wipe. One probe was the control probe to measure unstimulated sweating or trans-epidermal water loss. The second probe was placed over one of the tattooed measurement sites. To ensure accurate placement of the stimulated-site probe, we fabricated a clear plastic pattern with the exact outer dimensions of the probe head and an inner, 1 cm circle corresponding to the probe chamber with a point in its center. This was applied to the skin so that the center point was over the tattooed spot and its outline traced; the active probe was then positioned according to this outline. This ensured that the probe was precisely centered over the injection site and that the same areas would be measured with bubble and evaporimetry tests.

The measurement standard for TEWL used by the cyberDERM RG-1 evaporimeter is g/m^2^·hr on a scale from zero to 110. To facilitate comparison with the sweat bubble method we converted this to nl/(cm^2^·min) assuming 1 gram = 1 ml, so 1 g/(m^2^·hr) = 1.67 nl/(cm^2^·min). In the TEWL measurement guidelines [16], the average volar forearm measurement of TEWL for unstimulated skin was 5.6 g/(m^2^·hr) or 9.35 nl/(cm^2^·min). TEWL measurements combine transpiration of water vapor through the stratum corneum with evaporation of any liquid sweat secretion that occurs. Because each site on the skin has a different density of sweat glands and perhaps other properties affecting TEWL, we chose to use baseline values before and after stimulation as the best reference for measuring the magnitude of stimulated sweat. In fact, the two probes at different sites rarely differed by more than 1 g/(m^2^·hr) (1.67 nl/(cm^2^·min)). Because the tracing is updated more frequently than the values reported to Excel, we quantified stimulated TEWL using ImageJ. We used the freehand selections tool in ImageJ to outline and measure the area under the curve of probe B to the pre-stimulated baseline of probe B. The pixel measurements were calibrated by measuring the y-axis height in g/m^2^ and the x-axis distance in min using the straight lines tool, which gave the number of pixels in a unit of 1 g/(m^2^·h). The total pixel area was then expressed as g/(m^2^·h) and nl/(cm^2^·min).

### Stimulation

Because the main goal was to compare the sensitivity of the two methods, we omitted the methacholine injection and stimulated each site with a single intradermal injection of 0.1 ml of a β-adrenergic cocktail consisting of isoproterenol, aminophylline and atropine in lactated ringer’s. Our full strength cocktail has half the volume (0.1 ml) of the cocktail used by Sato [8], but 2X the concentration of active reagents, so that injected doses remain unchanged. Concentrations we use for 0.1 ml injections of full dose cocktail are 280 μM atropine, 160 μM isoproterenol, and 20 mM aminophylline. For reduced concentration cocktails, the atropine concentration was held constant and isoproterenol and aminophylline were reduced to establish the lowest level of responding seen reliably with either method.

After placement the probes were undisturbed for 2 min to establish baseline TEWL. Then the probe at the injection site was rotated 45 degrees around its long axis and moved away from the site, and the β-adrenergic cocktail was injected intradermally with a 30 gauge insulin syringe. The hypodermic needle was inserted just outside the outer wall of the probe and the tip advanced to just beneath the tattooed spot. The cocktail was injected over a period of ~15 sec, and the probe then replaced in alignment with the outline. After 20 min the probes were removed and placed in their cradles to establish 2 min of open-chamber probe baseline.

## Results

### Sweat gland populations and response properties at the four sites to full cocktail

When pre-stimulated with methacholine and then stimulated with the full β-adrenergic cocktail in separate experiments carried out previously for a different purpose, 40–71 glands were measured at each of the 4 sites (**Table 1**). Although our well is 1 cm^2^, the imaged area is only ~65 mm^2^, and ~5 mm^2^ is typically obscured by the measuring grid. Thus, gland densities for these areas ranged from ~0.7 to ~1.2 glands per mm^2^. The average total volumes measured after 20 or 30 min of stimulation are shown in **Table 1.** The gland numbers and total volumes produced in response to stimulation with full cocktail were used to calculate an approximate “% response” for the cocktails with reduced strength.

**Table 1:** Bubble imaging data for Subject EB01.

			Gland numbers		Gland volumes	
Test Date	Test Site	Cocktail conc.	Cocktail Glands (n)	% Full Cocktail	Cocktail Final Vol. (nl)	% Full Cocktail Vol.
	**L2**	**1**	**71**	**100%**	**794 +/- 54**	**100%**
2/26/2016	L2-1	0.001	39	54.9%	60.50	7.6%
3/11/2016	L2-2	0.001	40	56.3%	89.61	11.3%
4/14/2016	L2-3	0.001	39	54.9%	175.81	22.1%
**L2 averages**			**39.33**	**55.4%**	**108.64**	**13.7%**
	**L3**	**1**	**41**	**100%**	**592**	**100%**
2/26/2016	L3-1	0.001	19	46.3%	23.20	3.9%
3/11/2016	L3-2	0.001	26	63.4%	40.46	6.8%
4/14/2016	L3-3	0.001	20	48.8%	29.79	5.0%
**L3 averages**			**21.67**	**52.8%**	**31.15**	**5.3%**
	**R2**	**1**	**64**	**100%**	**824**	**100%**
3/4/2016	R2-1	0.001	13	20.3%	23.37	2.8%
3/18/2016	R2-2	0.001	29	45.3%	60.03	7.3%
4/21/2016	R2-3	0.001	23	35.9%	34.77	4.2%
**R2 averages**			**21.67**	**33.9%**	**39.39**	**4.8%**
	**R3**	**1**	**40**	**100%**	**581**	**100%**
3/4/2016	R3-1	0.001	18	45.0%	16.92	2.9%
3/18/2016	R3-2	0.001	24	60.0%	22.64	3.9%
4/21/2016	R3-3	0.001	22	55.0%	20.72	3.6%
**R3 averages**			**21.33**	**53.3%**	**20.09**	**3.5%**
3/25/2016	L2	0.0003	23	32%	18	2%
3/25/2016	L3	0.0003	5	12.2%	4.69	0.8%
4/7/2016	R2	0.0003	8	12.5%	9.73	1.2%
4/7/2016	R3	0.0003	1	2.5%	0.04	0.0%
**Averages at all sites**			**9.25**	**14.9%**	**8.13**	**1.1%**

The bold data in top row for each site L2-R3 shows gland numbers and total volumes secreted for that site based on averages from 3–12 experiments with full cocktail (100%) preceded by MCh. Gland numbers and total volumes for each of the tests with reduced cocktail are shown, and for each the "% full" represents the percentage of the average response to full cocktail. Total volumes are for 20 min at site L2 and 30 min at other sites, hence the "% of full cocktail” are underestimates for those sites.

**Fig 2:**
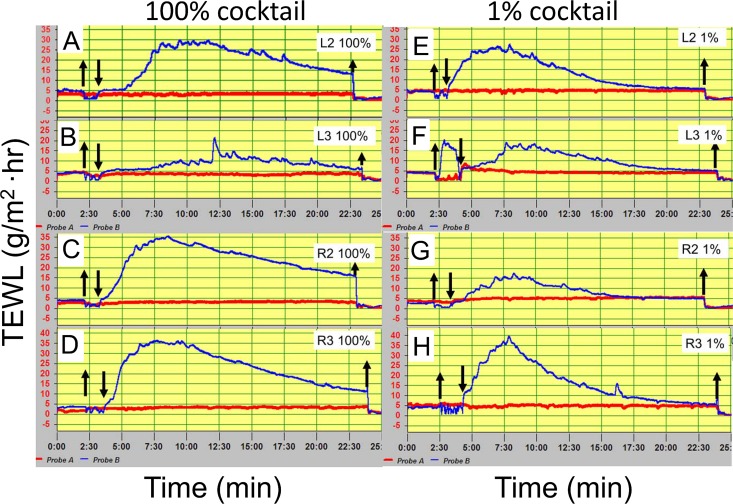
C-sweat responses of the heterozygote (EB01) to intradermal injections of 100% and 1% strength cocktail measured at 4 sites with evaporimetry. (A)-(D) show evaporimetry traces to the 100% cocktail strength at each of the 4 sites; (E)-(H) show traces to 1% cocktail. Down arrows show probes on and up arrows show probes off. Evaporimeter traces show TEWL units of 5 g/(m^2^·hr) (y-axis) and units of 2.5 min (x-axis).

Evaporimetry has been shown to produce reliable responses across multiple subjects when stimulated in this way [11]. Because our main interest was the sensitivity of the two assays, we did only a limited series of experiments at these sites using full cocktail strength with and without methacholine pre-stimulation. Responses to full cocktail had peak values of 20–35 g/(m^2^·hr) and calculated total volumes of 146 (L3) to 850 (L2) nl/(cm^2^·20 min) (**Fig 2A–2D** and **Table 2**).

**Table 2:** Evaporimeter measurements for Subject EB01.

		Diagnostic				TEWL (g/(m^2^·hr))			
Test Date	Test Site	Relative humidity (%)		Temperature (°C)		Base TEWL	Peak TEWL	Peak-Base	AUC (nl)
		upper	lower	upper	lower				
**Full Cocktail Concentration (1.000)**									
5/27/2016	L2	49.78	49.87	22.56	22.63	5	30	25	470.60
	Control	52.08	52.29	22.13	22.02	4	4	0	
6/2/2016	L2	55.44	55.77	22.49	22.57	4	45	41	850.00
	Control	56.76	57.2	22.34	22.25	4	5	1	
6/2/2016	L3	55.44	55.77	22.49	22.57	5	22	17	145.90
	Control	56.76	57.2	22.34	22.25	5	5	0	
5/27/2016	R2	49.78	49.87	22.56	22.63	4	35	31	651.09
	Control	52.08	52.29	22.13	22.02	4	4	0	
5/27/2016	R3	49.78	49.87	22.56	22.63	4	36	32	641.57
** **	Control	52.08	52.29	22.13	22.02	2	4	2	
**1% Cocktail Concentration (0.010)**									
4/29/2016	L2	41.33	41.26	22.77	22.88	5	27	22	261.08
	Control	42.88	42.91	22.61	22.53	5	5	0	
4/29/2016	L3	41.33	41.26	22.77	22.88	4	16	12	79.34
	Control	42.88	42.91	22.61	22.53	5	5	0	
4/29/2016	R2	36.51	36.07	22.44	22.52	3	17	14	101.66
	Control	38.57	38.84	22.8	21.97	4	5	1	
2/18/2016	R3	-	-	-	-	5	40	35	361.29
	Control	-	-	-	-	5	5	0	
**0.1% Cocktail Concentration (0.001)**									
3/4/2016	L2-1	57.37	57.75	22.93	23	4	20	16	49.35
	Control	57.03	57.32	23.13	23.03	4	4	0	
3/18/2016	L2-2	44.22	43.87	22.86	23.01	5	5	0	0.00
	Control	46.33	46.08	22.44	22.4	5	5	0	
4/21/2016	L2-3	53.81	53.62	21.63	21.73	4	4	0	0.00
	Control	56.41	56.27	21.14	21.07	4	4	0	
**Average**	**L2**	**52**	**52**	**22**	**22.58**	**4.3**	**9.7**	**5.3**	**16.45**
	Control	**53**	**53**	**22**	**22.17**	**4.3**	**4.3**	**0.0**	** **
3/4/2016	L3-1	57.37	57.75	22.93	23	4	7	3	27.83
	Control	57.03	57.32	23.13	23.03	3	3	0	
3/18/2016	L3-2	44.22	43.87	22.86	23.01	5	5	0	0.00
	Control	46.33	46.08	22.44	22.4	5	5	0	
4/21/2016	L3-3	53.81	53.62	21.63	21.73	4	4	0	0.00
	Control	56.41	56.27	21.14	21.07	3	3	0	
**Average**	**L3**	**52**	**52**	**22**	**22.58**	**4.3**	**5.3**	**1.0**	**9.28**
	Control	**53**	**53**	**22**	**22.17**	**3.5**	**3.7**	**0.0**	** **
2/26/2016	R2-1	47.63	47.41	22.16	22.25	4	4	0	0.00
	Control	48.99	48.88	21.99	21.9	4	4	0	
3/11/2016	R2-2	41.91	42.25	22.18	22.26	4	17	13	75.87
	Control	44.03	44.18	21.63	21.53	3	3	0	
4/14/2016	R2-3	36.67	36.57	21.44	21.52	5	5	0	0.00
	Control	38.54	38.57	21.12	21.03	5	5	0	
**Average**	**R2**	**42**	**42**	**22**	**22.01**	**4.3**	**8.7**	**4.3**	**25.29**
	Control	**44**	**44**	**22**	**21.49**	**4.0**	**4.0**	**0.0**	** **
2/26/2016	R3-1	47.63	47.41	22.16	22.25	5	6	1	0.00
	Control	48.99	48.88	21.99	21.9	4	4	0	
3/11/2016	R3-2	41.91	42.25	22.18	22.26	5	11	6	71.26
	Control	44.03	44.18	21.63	21.53	0	5	5	
4/14/2016	R3-3	36.67	36.57	21.44	21.52	5	5	0	0.00
	Control	38.54	38.57	21.12	21.03	5	5	0	
**Average**	**R3**	**42**	**42**	**22**	**22.01**	**5.0**	**7.3**	**2.3**	**23.75**
	Control	**44**	**44**	**22**	**21.49**	**3.0**	**4.7**	**1.7**	** **
**0.03% Cocktail Concentration (0.0003)**									
4/7/2016	L2	41.65	41.84	21.89	21.97	5	5	0	0.00
	C	44.50	44.73	21.26	21.16	5	5	0	
4/7/2016	L3	41.65	41.84	21.89	21.97	5	5	0	0.00
	C	44.50	44.73	21.26	21.16	5	5	0	
3/25/2016	R2	39.76	39.76	22.31	22.4	5	5	0	0.00
	C	41.22	41.41	22.07	21.97	4	4	0	
3/25/2016	R3	39.76	39.76	22.31	22.4	5	5	0	0.00
	C	41.22	41.41	22.07	21.97	4	4	0	

Data for 16 experiments is summarized. For each test the diagnostic measurements of probe relative humidity and temperature are shown for each probe, followed by TEWL measurements. The area under the curve (AUC) gives estimated total volume for stimulated secretion converted to nanoliters (see text). The area under the curve (AUC) gives estimated total volume for stimulated secretion converted to nanoliters (see text).

### Evaporimeter responses to 1% cocktail

A previous study with sweat bubble imaging using the same CF carrier as these studies showed that injection with a β-adrenergic cocktail at 1% normal strength and without prior potentiation via methacholine injection produced detectable responses in ~70% of the glands that responded to full cocktail (potentiated responses were increased ~140%, n = 6) [10]. For comparison, we measured evaporimetry responses to a 1% cocktail stimulus and detected clear responses at all 4 sites (**Fig 2E–2H** and **Table 2**). Responses measured (AUC-baseline) were 55–70% of full cocktail with one outlier at 9%; they had lower peaks and faster declines than the responses to full cocktail.

### Sweat bubble and evaporimeter responses to 0.1% cocktail

**Fig 3:**
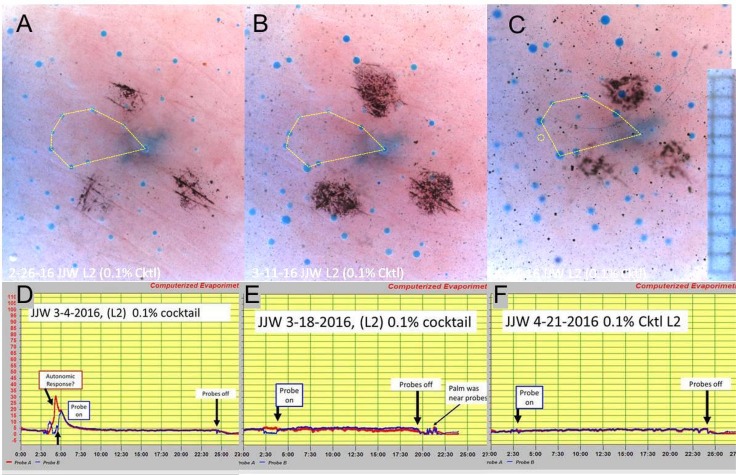
C-sweat responses at site L2 to injection of 0.1% strength cocktail measured with the bubble method and with evaporimetry. Each panel A-C shows bubble responses. (A). For this and following images, the fuzzy dark spot in the center is a tattoo to mark the spot, and the 3 surrounding dark spots are ink marks to aid focus and orientation. Eight glands are connected by the yellow dotted line to illustrate how constellations of identified glands can be followed across tests. (B) Same site as A, two weeks later. (C) Same site as A, 4 weeks later. This was the largest sweat bubble response seen out of 12 tests. Here, G93 in the marked constellation did not secrete (dotted circle) even though overall secretion was higher on this test. Grid squares on 0.5 mm. D-F show evaporimetry responses at the same site, tested one week later in each case. Probe A (red traces) are the control (not-injected site) and probe B (blue traces) are the injected site. (D). Large transient responses occurred at both sites at the time of the injection. The subject expressed alarm because the injection needle appeared close to a vein. (E, F): As for D, but 2 and 6 weeks later.

**Fig 4:**
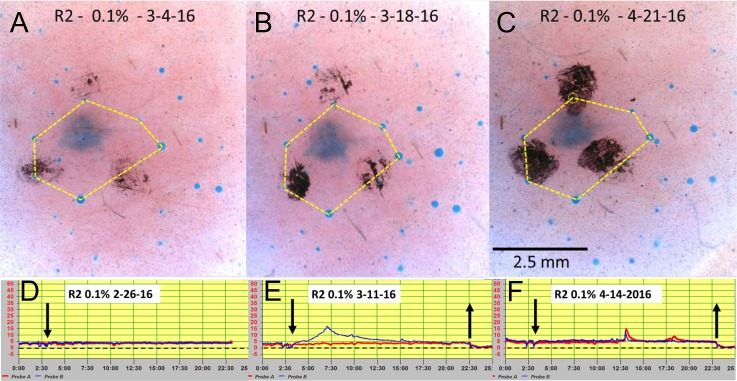
C-sweat responses at site R2 to injection of 0.1% strength cocktail measured with the bubble method and with evaporimetry. This site was chosen because it showed the largest evaporimetry response seen to 0.1% cocktail. (A-C) show bubble responses, the same 6 glands were connected by thin yellow lines in each image except for (C), where no bubble was visible at the apex of the hexagram (dotted circle). (D-F) show evaporimetry responses at the same site offset by one week. All other descriptors are as for Fig 2. The largest evaporimetry response to 0.1% cocktail is shown in (E). Excursions in (F) are artifacts of unknown cause. Down arrow: probe B on; up arrow: probes off.

Prior dose-ranging studies indicated that bubble responses could be obtained at cocktail levels 3 log units below the standard full strength (Figure 7 in ref [10]), so we used sweat bubble imaging to measure responses to 0.1% cocktail at each of the 4 sites, with three tests at each site. We tested the same 4 sites with evaporimetry on alternate weeks, again with three tests at each site. The bubble responses and corresponding evaporimetry responses are shown for two of the four sites in **Figs 3 and 4**. For each figure, the top 3 images (A-C) show the bubble responses and the bottom three (D-F) show corresponding evaporimeter traces, displaced by one week. Site L2 data are shown because this site produced the highest aggregate sweat volume measured with bubble imaging. Site R2 data are shown because the second R2 test produced the largest evaporimeter response we saw. When they were detectable, TEWL responses were transient (**Fig 4E**), and their computed aggregate volumes were smaller than the aggregate TEWL baseline of 134–167 nl/cm^2^ for the 20 min measurement period.

**Fig 5:**
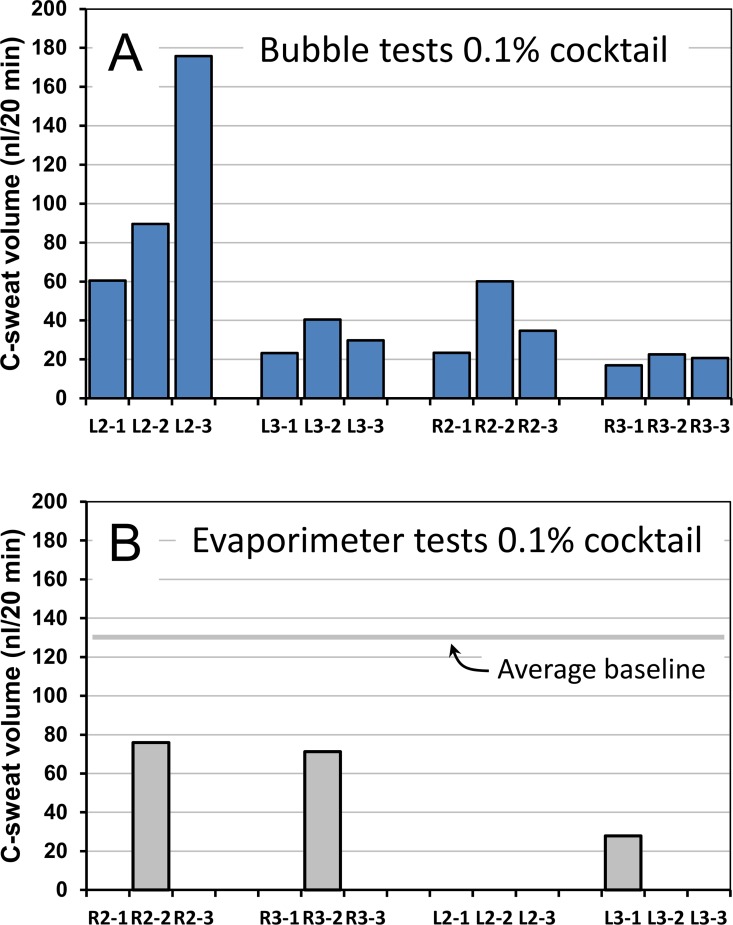
Summary of responses to 0.1% cocktail in a CF carrier measured in triplicate at 4 sites with sweat bubble imaging and evaporimetry. (A) Total volume of sweat imaged after 20 min stimulation at each site (blue columns). (B) Total volume of sweat estimated from evaporimetry measurement of TEWL (see text). The average TEWL baseline for this subject across all 12 tests is shown; this value was subtracted from the transient sweat responses (gray columns).

The 0.1% level of stimulation discriminated well between the two assays (**Fig 5**). Bubble imaging detected quantifiable responses for 12/12 (100%) of the tests (**Fig 5A**), with total volumes per test/per site that ranged from ~2.8% to 22% of the volume secreted to full cocktail at the corresponding sites (**Table 1**). L2 was the most responsive site, with 39/71 (55%) of glands responding to produce on average 14% of the volume of sweat observed with full cocktail. Using evaporimetry, only 3/12 tests (25%) produced quantifiable, stimulus-dependent TEWL responses (**Fig 5B)**.

### Sweat bubble and evaporimeter responses to 0.03% cocktail

**Fig 6:**
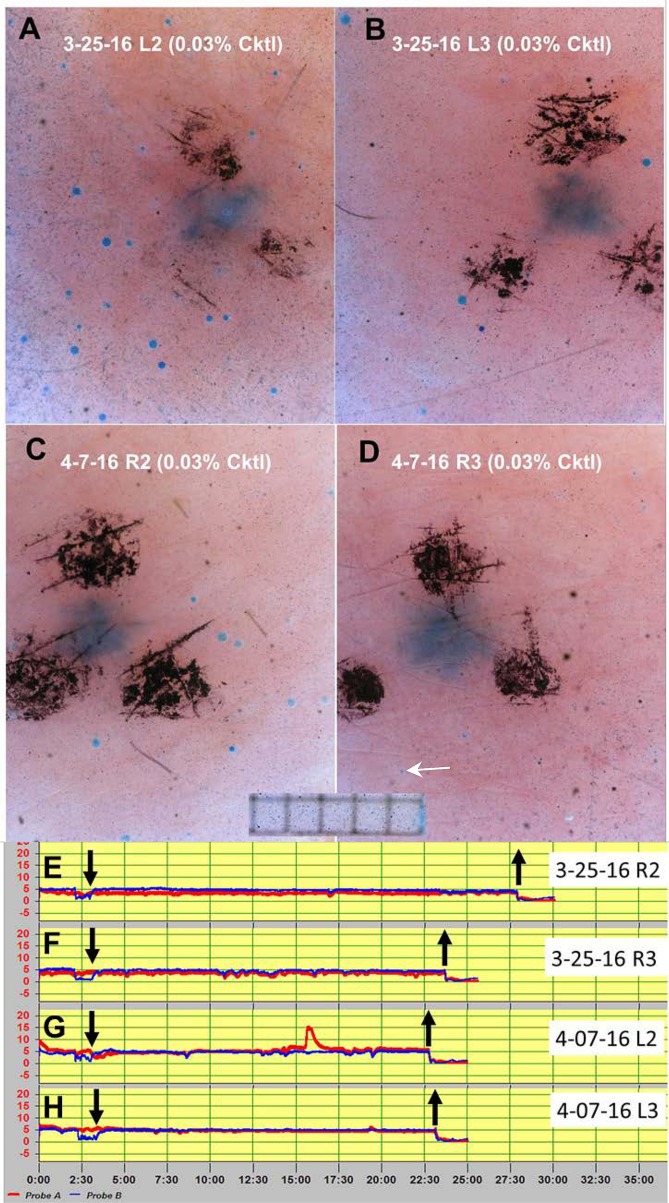
Responses to 0.03% cocktail in a CF carrier measured once at the same 4 sites with sweat bubble imaging and evaporimetry. (A-D) show sweat bubbles after 20 min of stimulation with 0.03% cocktail strength at each of the 4 sites (cropped images). (E-H) show evaporimetry traces to the 0.03% cocktail strength at each of the 4 sites. Down arrows show probes on and up arrows show probes off. Grid squares in (A-D) are 0.5 mm. Evaporimeter traces (E-H) show TEWL in units of 5 g/(m^2^·hr) on y-axis and units of 2.5 min on x-axis.

To probe the threshold of the two assays, we made a further reduction in cocktail strength to 0.03% and tested each of the 4 sites once with bubble imaging and once with evaporimetry. C-sweat responses from multiple glands were observed at 3/4 sites (**Fig 6A–6C**) and a single gland was seen to respond at site R3 (**Fig 5D**). No responses were detected by evaporimetry to the 0.3% cocktail concentration (**Fig 6E and 6F, Table 1**).

### Sweat bubble and evaporimeter responses in the absence of stimulation

It has long been known that sweating contributes to TEWL in a minority of subjects because their TEWL levels can be lowered by anticholinergics. For example, in 44 subjects tested before and after prolonged treatment with topical scopolamine, TEWL values were unchanged in 38 subjects (86%), but were lowered by 1–1.8 g/(m^2^·hr) in 6 subjects (14%) [17]. Subject EB01 had a low resting TEWL level that was not affected by a high concentration of atropine injected intradermally, as can be seen by the return of the baseline to pre-injection levels after the effects of the β-adrenergic stimulating agents in the cocktail had waned (Fig 2D–2F and Fig 3D–3F and Fig 5E–5H), strongly suggesting that this subject did not produce a measurable amount of basal sweat secretion.

**Fig 7:**
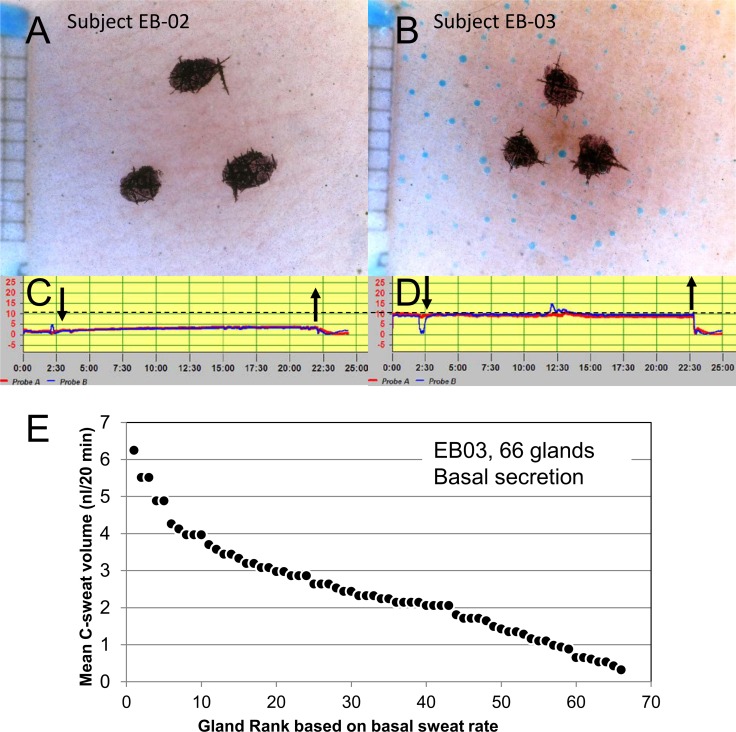
Bubble imaging and TEWL measures with evaporimetry in two subjects (EB-02, 03) who were not stimulated. (A-B) show sweat bubble imaging after 20 min with no applied stimulus (and with no atropine to block any baseline cholinergic secretion). (C-D) show evaporimetry traces at the same sites. The dashed line shows a stable and consistent difference between the two subjects of ~6 g/(m^2^·hr) or 10 nl/(cm^2^·min). The summed bubble secretion corrected for the 0.6 cm^2^ size of the imaged area was 13.4 nl/cm^2^ ·min. This agreement is at least as good as the agreement for bubble tests at same sites on different days shown in Fig 5. However, in subsequent tests with this subject no bubbles were observed in spite of the same TEWL (see text). (E) Distribution of single gland basal secretion rates for sweat bubbles (n = 66) shown in panel (B). Each point plots the volume (y-axis) and rank for an individual gland.

Because this study is the first to measure sweating in subjects with both evaporimetry and bubble imaging, we felt it would be useful to compare the methods in subjects with higher and lower TEWL values to see if bubble imaging could also discriminate between them. By chance the first two subjects tested with evaporimetry in the absence of stimulation had substantially different TEWL levels. On three separate days Subject EB02 had TEWL measurements of 2.7 ± 1.5 (Probe A) and 2.3 ± 1.5 g/(m^2^·hr) (Probe B), while on 4 separate days EB03 had TEWL measurements of 10.0 ± 0.8 (Probe A) and 9.8 ± 0.5 g/(m^2^·hr) (Probe B); see **Fig 7**. We then performed bubble imaging without stimulation at the same probe B sites used for evaporimetry. For EB02 we ran two imaging tests–no sweating was seen in either one (**Fig 7A**). For EB03 we ran 3 tests. No sweating was seen in 2 of 3 tests, but substantial sweating was seen during one test (**Fig 7B**). For that single test, 66 sweat glands produced 160.8 nl of sweat during the 20 min observation period, with sweat production per gland varying over an almost 20-fold range (Fig 7E). We do not know what factors differed prior to the positive test, and because of the way the tests were done we did not have parallel evaporimetry data.

Our interpretation of these results is that baseline TEWL in the three subjects primarily represents exit of water vapor across the skin, but that on some occasions basal sweating can occur. On the one occasion when basal sweating did occur, rates were stable across the 20 min period, unlike the transient increases produced by injected cocktail, and this stability simplified the comparison of TEWL and bubble measures of sweating in EB03. The imaged bubble secretion (**Fig 7B**), corrected for the 0.6 cm^2^ size of the imaged area, gave a rate of 13.4 nl/(cm^2^·min). The average TEWL for this subject was 16.7 nl/(cm^2^·min). If our interpretation is correct, it predicts that evaporimeter probes would have measured a TEWL of almost 30 nl/(cm^2^·min) or 18 g/(m^2^·hr) if they had been on this same site at the same time.

### Gland-by-gland analyses

To this point, we have compared bubble imaging and evaporimetry simply by summing the volumes produced by all imaged glands or by integrating the evaporimeter response. However, gland-by-gland analysis, available only with the bubble imaging method, can provide additional advantages [10]. **Fig 7E** shows the distribution of basal secretion volumes at the 20 min time point for the 66 individual glands that secreted spontaneously in subject EB03. Gland density is ~ 1 gland/ mm^2^, similar to stimulated sites and suggesting that most glands at the site were secreting basally. The volumes ranged from 0.32 to 6.25 nl, reflecting a nearly 20-fold range of average from 0.016 to 0.31 nl/(gl·min).

**Fig 8:**
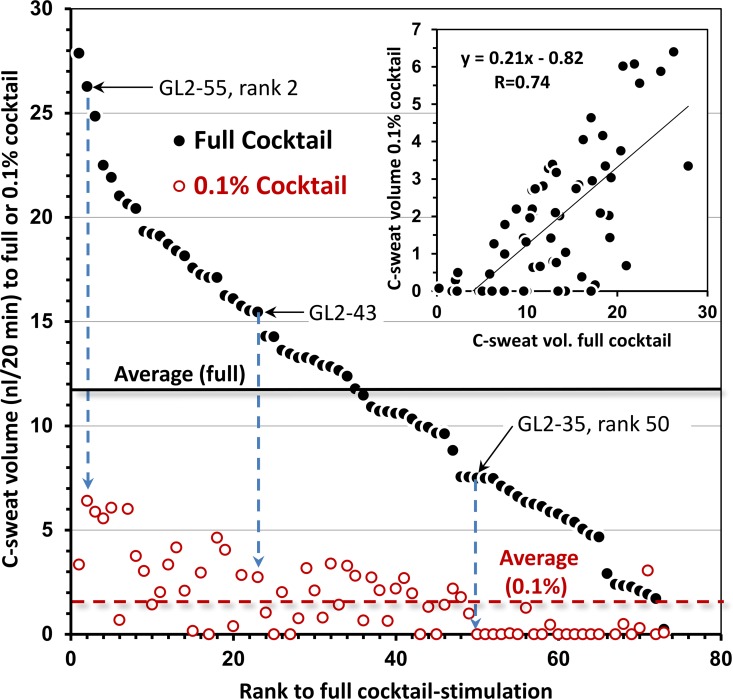
Distribution of responses to two cocktail strength for 73 identified glands at site L2 of subject EB01. Glands are rank ordered by the volume of their C-sweat in response to full cocktail (filled circles), and the corresponding response of each glands to the 0.1% b-adrenergic cocktail is shown directly below (open circles). Each pair of points represents the mean total volumes secreted during 20 min across 1–3 trials at the two cocktail strengths. (Three selected pairs of responses are connected by vertical arrows and are labeled by gland ID and rank based on response to full cocktail.) The solid horizontal line is the mean response to full cocktail and the dashed horizontal line is the mean response to 0.1% cocktail. The average coefficient of variation for individual gland responses was 0.36 to the full cocktail and 0.90 for 50 of 73 glands that responded to the 0.1% cocktail on at least one trial. Inset plots correlation of the two responses; R = .74, P <0.001.

For a further gland-by-gland analysis we selected site L2 from subject EB01. In **Fig 8**, 73 identified glands at site L2 are rank ordered along the x-axis according to the mean volume (3 tests) of C-sweat produced over 20 min to full cocktail (y-axis, solid points). The plot illustrates the wide (> 15-fold) range and near continuous variation of responses observed for stimulated C-sweat, similar to the basal secretion seen in Fig 7E. For each gland the corresponding mean responses to 3 tests with 0.1% cocktail (open points) are aligned as shown by the dashed arrows for 3 of the labeled glands. The two responses are correlated (inset, R = 0.74, P <0.001). One third of the glands did not respond to the 0.1% cocktail on any of three trials, with the probability of failure increasing as secretion rates to full cocktail decreased, such that most glands with a secretion rate to full cocktail ≤0.375 nl/min failed to produce measurable sweat to the 0.1% cocktail. The implications of this for assessment of CFTR modulators that produce small increases in CFTR function are discussed below.

## Discussion

### Bubble imaging is more sensitive than evaporimetry

The main finding is that bubble imaging is more sensitive than evaporimetry. While each method reliably detected C-sweat rates produced by 1% cocktail, only bubble imaging reliably detected responses to 0.1% cocktail (Fig 5). For the 0.1% cocktail, aggregate sweat volumes detected by bubble imaging on individual tests with EB01 were 2.8–22% of maximal C-sweating at the same sites. Evaporimetry at these sites detected responses on only 3/12 tests. At 0.03% cocktail evaporimetry did not detect sweating, while aggregate sweat volumes detected by bubble imaging were ~1% of the response to full cocktail.

### Bubble imaging confirms that in some subjects basal sweating can contribute to TEWL

A secondary finding is confirmation that sweating sometimes contributes to resting (unstimulated) TEWL for a minority of subjects [17]. Two subjects had stable basal TEWL levels < 5 g/(m^2^·hr) and in these subjects no basal sweating was observed with bubble imaging. For a third subject TEWL was consistently 10 g/(m^2^·hr) and bubble imaging detected basal sweat secretion on 1 of 3 tests. Further evidence that TEWL of 5 g/(m^2^·hr) does not involve sweat secretion is that all of our cocktails, which contained the same high level of atropine (1.4 x 10^−4^ M) that completely blocks sweating to high dose methacholine (5 x 10^−4^ M) [8], did not lead to any diminution of the TEWL response even with cocktails having the lowest concentrations of β-adrenergic components.

### Why is bubble imaging more sensitive?

The evaporimeter measures a skin area ~1 cm^2^ and the smallest amount reliably measured is reported to be 2.16 g/(m^2^·h) [16] or 3.6 nl/(cm^2^·min). This estimate fits well with what we observed. Although all 12 bubble imaging experiments detected clear C-sweating to 0.1% cocktail, only 2 of 12 had aggregate rates >3.6 nl/(cm^2^·min) (72 nl/20 min), Fig 5A. Consistent with that being a lower limit for evaporimetry, parallel experiments with evaporimetry detected sweating on only 3 of 12 experiments (Fig 5B). Several features distinguish the two methods. First, evaporimetry measures the gradient of water vapor concentration near the skin, which is determined by the rate of water vapor production. This rate is in turn made up of ‘insensible’ water vapor loss across the skin (which is what TEWL was designed to measure), but also to vapor from evaporating liquid sweat (the goal of CF-related evaporimetry studies). At low rates of vapor production, random thermal motion eliminates the gradient, producing equivalent readings at the two sensors. By contrast, water vapor is invisible to bubble imaging. Second, evaporimetry measures the rate of loss of the sweat sample and hence is a destructive assay, whereas bubble imaging traps the sweat as it emerges from each gland, allowing even extremely low secretion rates to accumulate over time into detectable volumes. (The preserved sweat can then be used for chemical analyses, or even for volume measurement via other methods.) Third, bubble imaging focuses on the target source of sweat secretion: the sweat duct openings. Setting the diameter of sweat duct openings to 50 μm gives a ductal opening area = 1963.5 μm^2^. For subject EB01 we saw < 1 gland/mm^2^ so sweat duct openings constituted <0.2% of the skin area. This 500-fold disparity helps explain why the low rate of insensible water vapor loss can equal or exceed low sweat rates.

### Advantages of identifying individual glands

To compare the two methods we used aggregate sweat rates from the measured skin sites, but the bubble imaging assay was designed to quantify secretion rates for individually identified glands so that they could be compared across experiments, thus converting a single measure into a sample with n ≈ 50 distinctive, semi-independent measures [10]. Sweat glands display a wide range of secretion rates both basally (Fig 7) and to intradermal injections (Fig 8). The wide range of rates (almost 20-fold) for basal secretion demonstrates that these are inherent to the glands, and do not primarily result from the distribution of injected agonists within the intradermal space. The range of rates is expected to mainly reflect differences in gland size, but the only data on gland size we could find indicates a more restricted range of 2.5 to 6.3-fold [18]. If the gland size data are accurate, other factors must play a role. We do not know what these might be, but the range of rates is seen for both cholinergic and β-adrenergic stimulation, and these rates are highly correlated [6].

In Fig 8, the open points plotting responses to 0.1% cocktail are considerably larger than the low secretion rates produced in G551D subjects taking ivacaftor [6]. Even so, 23/73 (31%) of the responses to 0.1% cocktail fell below the limit of detection with bubble imaging (across 3 tests), and ~54% were undetected on any single test. Thus the gland-by-gland analysis reveals, in a single individual, the dependence of detectable responses on gland size. It therefore provides a principled way to assess CFTR function across individuals by selecting a set of glands matched by a surrogate for gland size—namely the M-sweat rate that is relatively independent of CFTR function [6]. Clearly, selection of the largest glands increases the sensitivity of the assay.

It is important to identify glands that fall below the threshold of detection so that they can be dealt with separately in the analysis. A separate question is why we fail to see secretion by these glands. The possibilities are that 1) the glands are not secreting, 2) they are producing secretion at a rate that is below the resolution of the present bubble-imaging assay, or 3) they are secreting at a rate that could be detected, but the secreted fluid is lost before reaching the surface because the duct lumen needs to be filled and some fluid is absorbed [6, 10]. We favor the last possibility, which is consistent with earlier findings by Shamsuddin *et al*., who compared responses of sweat gland cells in isolated sweat coils and responses of intact glands. Used alone, isoproterenol clearly stimulated secretion in the isolated coil, yet no sweat secretion was seen in the intact gland unless the stimulus was enhanced with a phosphodiesterase inhibitor [19].

### Limitations of the study

Several features of this study were non-optimal. The cocktail concentrations were not administered in randomized order, and a full dose-response relationship was not attempted. Neither the operators nor the subject were blinded to the concentrations being used. The use of a single subject would typically be cause for concern, but the object here was to test the instruments, not the subject. Therefore, the more pertinent limitation is that only one evaporimeter and one bubble-imaging set-up were compared, and in only one laboratory.

## Conclusions

Because of the advantages outlined in the introduction, the eccrine sweat gland is exceptionally well-suited for reporting the level of CFTR function in human subjects. Multiple assays have been developed to exploit these advantages, and each has its own set of advantages and disadvantages. Some advantages of the bubble imaging method were outlined previously [6, 10]; the present results quantify its sensitivity relative to evaporimetry and illustrate some additional insights made possible by the ability to follow the function of individually identified glands.
